# Digital Nutrition Care Ecosystem: Integrating Critical AI Nutrition Literacy, Countering Nutrition Misinformation, and Dietitian-Led Care

**DOI:** 10.3390/nu18213663

**Published:** 2026-11-04

**Authors:** Wen-Chin Yeh, Chung-Mei Ouyang, Yau-Jiunn Lee, Hui-Chun Hsu

**Affiliations:** 1 Department of Clinical Nutrition, Lee’s Clinic, 130 Min-Zu Rd., Pingtung 90000, Taiwanmaggieoy@gmail.com (C.-M.O.); 2 Department of Internal Medicine, Lee’s Clinic, 130 Min-Zu Rd., Pingtung 90000, Taiwan; 3 Department of Nursing, Lee’s Clinic, 130 Min-Zu Rd., Pingtung 90000, Taiwan

**Keywords:** digital nutrition education, critical AI nutrition literacy, nutrition labels, nutrition misinformation, digital nutrition care ecosystem, clinical dietitian, human-in-the-loop, Nutrition Care Process

## Abstract

Chatbots and digital tools can make nutrition information more accessible, yet fluent explanations and precise-looking nutrient estimates may conceal unverified assumptions, portion-size errors, and inconsistent outputs. However, few frameworks connect critical appraisal of such outputs with routine clinical nutrition workflows and education. Building on a comparison of dietitian and chatbot interpretations of ready-to-eat meal labels, we propose the Digital Nutrition Care Ecosystem (DNCE), a human-supervised, Nutrition Care Process-aligned framework that links clinical nutrition workflows with critical AI nutrition literacy. DNCE connects eight functions: data acquisition, data integration, intelligence, nutrition diagnosis and clinical decision-making, individualized medical nutrition therapy, food implementation, delivery and engagement, and monitoring and learning. Within this ecosystem, dietitians are not replaced: repetitive, reversible, verifiable tasks may be supported by automation, whereas interpretation, individualized decisions, counseling, and final authorization remain professionally supervised. The paper contributes a proposed five-question educational checklist addressing (1) information source, (2) serving size, (3) assumptions and uncertainty, (4) consistency and verification, and (5) escalation before consequential action. Expected benefits include better recognition of unsupported nutrition claims, appropriate help-seeking, less correction work, and more dietitian time for judgment-intensive care. DNCE and the checklist are conceptual proposals that require empirical validation.

## 1. Introduction

Digital nutrition has progressed from electronic food records and tele-nutrition toward multimodal dietary assessment, automated documentation, artificial intelligence (AI)-supported personalization, and clinical decision support [1,2,3]. The implementation challenge is no longer whether a digital tool can perform an isolated task; it is whether multiple tools can be organized around professional nutrition care in a way that reduces low-value workload, improves continuity and safety, and strengthens the effective use of dietitian time. A recent scoping review of AI-supported dietetic practice found applications across several stages of the Nutrition Care Process (NCP), while also emphasizing variable accuracy, patient-safety concerns, and the need for professional training and oversight [3].

A second challenge is educational. Chatbots and other generative AI systems increasingly provide nutrient estimates, interpret nutrition labels, and generate dietary explanations directly for students, patients, consumers, and health professionals. These tools can make nutrition information easier to access, but convenient output can be mistaken for verified fact. In the E+DIEting_Lab evaluation of 475 nutrition and dietetics students, 79.7% reported improved practical skills, and 90% felt that new digital tools improved classroom practice; post-use ratings also suggested that hands-on experience refined initial expectations rather than simply increasing enthusiasm [4]. At the same time, nutrition and food literacy research emphasizes that people need skills not merely to obtain information, but to judge its source, context, credibility, and applicability [5]. Digital communication by nutrition professionals may help counter misinformation, but it also requires training in content validation and ethical communication [6].

Clinical dietitians therefore face two connected educational and clinical responsibilities: helping people interrogate chatbot-generated nutrition claims, while using digital tools responsibly within the Nutrition Care Process. Responding to a need highlighted by the variability of AI-derived nutrient estimates, we propose the Digital Nutrition Care Ecosystem (DNCE) as a human-centered conceptual framework that links critical AI nutrition literacy to professional reasoning, interoperable data, individualized medical nutrition therapy (MNT), real-world food implementation, and longitudinal evaluation. In this Perspective, we define critical AI nutrition literacy as the ability to distinguish source-grounded nutrition information from model inference, recognize assumptions and uncertainty, verify consequential claims against authoritative sources, and determine when professional review is required before action. Its central educational question is not simply whether a chatbot can provide an answer, but whether a learner can determine when that answer is grounded, uncertain, or unsafe to act upon.

Despite the increasing adoption of digital tools in nutrition care, integrated frameworks that connect critical AI nutrition literacy with daily clinical practice and education are still lacking. DNCE aims to fill this gap by linking data capture, verified calculation, AI-supported interpretation, and professional judgment within a closed care loop, and by making the reliability of each piece of nutrition information visible to the people who use it. A central element of this framework is making the distinction between source-grounded nutrition data and model-inferred values explicit and teachable, so that learners and professionals can identify, verify, and act appropriately on nutrition information. If implemented, DNCE is expected to improve the ability of dietitians and learners to critically appraise AI-generated nutrition information, enhance patient safety, and optimize the use of professional time; these expectations are hypotheses to be tested (Section 10).

## 2. Perspective Development and Scope

This article is a Perspective, not a systematic or scoping review, and does not report a new educational trial. It selectively synthesizes established nutrition-care frameworks, digital-health standards, recent literature on AI-supported dietetics and clinical documentation, and a previously published nutrition-label comparison conducted by a Taiwan-based clinical team [7]. These sources inform a proposed framework and illustrate plausible mechanisms and implementation risks; literature selection was narrative rather than systematic. A narrative synthesis was chosen because the aim was to integrate concepts across fields with heterogeneous evidence bases—dietetics, clinical informatics, AI evaluation, health-system governance, and education—rather than to estimate an effect within a single, well-defined body of evidence, for which a systematic review would be appropriate. No new participant-level data were generated or analyzed, and neither DNCE nor the proposed education checklist has been prospectively validated. Accordingly, the Perspective covers conceptual synthesis, an illustrative AI architecture and governance model, a proposed educational checklist, and plausible mechanisms; it does not provide empirical validation, new data collection, an exhaustive literature review, or evaluation of specific commercial products.

During manuscript development, generative AI was used for structural editing, language refinement, preliminary conceptual visualization, and assistance in identifying potentially relevant literature. All candidate sources were independently reviewed, verified, and selected by the authors. Every reference was checked against its digital object identifier or bibliographic database record, and all AI-assisted text was rewritten or approved by the authors. Generative AI was not used to generate study data or make final scientific or clinical judgments. Final figures were redrawn under author control. Product details and the required disclosure are provided in the Acknowledgments section.

Because DNCE and the checklist are conceptual, their value must be established empirically. Section 10 therefore outlines a phased research plan that progresses from expert review and usability testing to technical benchmarking, feasibility studies, and controlled comparisons with usual nutrition education and existing care workflows.

## 3. Why an Ecosystem Rather than Isolated Digital Tools?

The NCP already provides a rigorous sequence of nutrition assessment, nutrition diagnosis, nutrition intervention, and nutrition monitoring and evaluation; standardized terminology supports structured documentation of that process [8]. Learning health system thinking frames evidence generation and application as natural products of care itself [9]. The Chronic Care Model emphasizes prepared care teams and informed patients [10], while Food Is Medicine and teaching-kitchen approaches connect clinical recommendations with food access, preparation, and real-world implementation [11,12,13]. DNCE therefore does not claim novelty for cyclic care, multidisciplinary management, patient-centeredness, or feedback alone. Nor is DNCE a new predictive or generative algorithm; its contribution lies in how existing computational components are orchestrated, constrained, and supervised in nutrition care. Table 1 compares DNCE with existing categories of AI-supported nutrition tools.

DNCE addresses four linked gaps, with education connecting them. First, prescription-to-plate implementation recognizes that an appropriate recommendation has limited effect until it becomes obtainable, prepared, acceptable, and consumed food. Second, nutrition-specific semantic and decision interoperability requires intake, nutrition diagnosis, prescriptions, nutrient constraints, food products, and outcomes to remain computably connected. Third, workflow orchestration should reduce duplicate entry and correction burden rather than add parallel systems. Fourth—and especially pertinent to digital nutrition education—interpretation-to-literacy translation makes the distinction between label-extracted facts and model-inferred values visible and teachable, so that learners can question assumptions, verify high-consequence information, and seek a dietitian’s review when needed. The first three functions create the clinical and informational context in which the fourth can be delivered responsibly.

Real-world experience illustrates why these gaps matter. In 2023, a United States eating-disorder support organization withdrew its chatbot after users reported calorie-deficit and weight-loss advice; the tool had been built on scripted responses, but a generative feature had reportedly been added by the vendor without the organization’s approval [14]. Isolated tools can also produce unstable numbers: in a comparison of ready-to-eat meal boxes, the coefficient of variation for sodium across repeated queries of the same image reached 69.2% for some AI models [7], and image-based estimation by a general-purpose model improved only when ingredient and preparation context was supplied [15]. In the authors’ clinical experience, dietary records kept in consumer apps or as photographs are frequently reviewed as screenshots or printouts and then re-entered manually. DNCE addresses these problems through version control and risk-tiered review (Section 9), provenance labels that separate source-grounded from model-inferred values (Section 5), and interoperable data flow that avoids duplicate entry (Section 4).

These mechanisms generate hypotheses rather than demonstrated outcomes: explicit food implementation may improve prescription-to-consumption fidelity; semantic interoperability may reduce missing-information and reconciliation errors; risk-tiered human–AI collaboration may improve clinical safety; coordinated automation may increase net effective dietitian capacity; and a structured, label-centered AI-literacy intervention may improve detection of unsupported or inconsistent outputs and appropriate verification behavior. Evaluation should test these propositions against usual nutrition education and well-implemented existing care processes.

## 4. WHO-Aligned Interoperability, Governance, and Workforce Capacity

The World Health Organization (WHO) provides a broader digital-health context. Its Global Strategy on Digital Health 2020–2027 emphasizes person-centeredness, governance, equity, interoperability, privacy, security, workforce capacity, and sustainability [16]. WHO–International Telecommunication Union guidance similarly treats digital platforms as infrastructure for integrated, interoperable health systems [17]. DNCE makes workforce capacity explicit: the purpose of digital infrastructure is not merely to move data, but to allow qualified nutrition professionals to spend more time on work that requires judgment, communication, and therapeutic relationship.

Nutrition makes interoperability concrete. HL7 FHIR NutritionIntake can represent consumed food or fluid, while NutritionOrder can represent diet, supplement, or enteral nutrition requests [18,19]. The WHO SMART Guidelines provide a complementary standards-based approach for translating recommendations into interoperable digital components [20]. When information can be reused across assessment, diagnosis, intervention, education, and monitoring, the dietitian spends less time re-entering information and more time interpreting it with the patient.

A brief scenario illustrates these benefits. A person with type 2 diabetes and chronic kidney disease photographs meals and records glucose at home. With interoperable data standards, meal records, glucose summaries, and the latest laboratory results arrive in one record, so that the dietitian does not re-enter data. A rule-based check flags that recent sodium intake exceeds the individual target. Speech-to-text and a language model draft the counseling note in standardized Nutrition Care Process terminology, which the dietitian reviews and signs. Time previously spent on transcription and data reconciliation can then be redirected to counseling on food choices and cooking methods. Governance contributes by registering each AI model and prompt version, reviewing changes before deployment, and making every AI-assisted recommendation traceable to its sources and reviewing professional.

Implementation challenges are nonetheless substantial. Technically, many electronic health records lack structured nutrition fields, and local foods are not always mapped to food-composition databases; FHIR-based exchange, local-to-national food mapping, and phased implementation can mitigate these gaps. Regulatory challenges include personal-data protection and uncertainty over whether AI functions qualify as medical devices; privacy-by-design, data minimization, early consultation with regulators, and classification of functions by risk tier are appropriate responses. Equity barriers include limited digital literacy among older adults and uneven connectivity, which call for assisted or hybrid modes, caregiver involvement, low-bandwidth options, and stratified evaluation. Financing is a further barrier because digital nutrition care is rarely reimbursed; embedding DNCE functions within existing chronic-disease programs and collecting cost data from pilots may help. Finally, workforce capacity depends on training dietitians and educators in critical AI nutrition literacy and on clearly defined professional roles in each risk tier.

## 5. The DNCE Closed-Loop Care Cycle

DNCE is organized as eight components of a closed-loop care cycle (Figure 1), not a mandatory technology stack. Governance, interoperability, standards, privacy, security, workforce capacity, and sustainability constitute its enabling environment. Person-centered engagement, ethics, equity, risk-tiered professional oversight, and workflow efficiency operate across the cycle. Critical AI nutrition literacy is represented in Figure 1 as a cross-cutting capability rather than as a ninth component of the DNCE cycle. Component names are used identically in the text, Figure 1, and Table 2.

Data acquisition captures diet history, food images, patient-reported outcomes, device data, and contextual information. Data integration connects electronic health record information, laboratory values, medications, social determinants of health, nutrition intake, and standardized semantics. Intelligence applies AI or machine learning, natural language processing, computer vision, retrieval-augmented generation (RAG), prediction, and uncertainty handling. Nutrition diagnosis and clinical decision-making remain anchored in professional reasoning and standardized nutrition terminology. Individualized MNT specifies nutrient, food, behavioral, and monitoring targets. Food implementation translates prescriptions into obtainable, preparable, and culturally acceptable food. Delivery and engagement support education, tele-nutrition, coaching, shared decision-making, and family or community support. Monitoring and learning return nutritional, clinical, patient-reported, workflow, educational, quality, utilization, and cost outcomes to earlier components so that care and education can adapt over time.

Within the intelligence component, DNCE assigns each AI technique a bounded role across four functional tiers. In the capture-and-structuring tier, computer vision identifies foods and estimates portions from images, speech-to-text transcribes recalls and consultations, and natural language processing, including large language models (LLMs), extracts structured fields from free text and label images; these outputs are candidate data that require confirmation. In the deterministic-calculation tier, nutrient totals are computed from verified sources, namely national food-composition databases and manufacturer labels, and rule-based checks compare intake with the individual prescription; generative models may call these functions but may not replace them. In the prediction tier, validated machine learning models estimate risks or responses, such as postprandial glucose excursions; these outputs are probabilistic and require separate validation. In the generative-interpretation tier, an LLM using RAG over a curated, version-controlled corpus drafts explanations, education materials, and summaries for professional review, cites retrieved sources, and cannot override calculated values.

Every value shown to a user therefore carries one of three provenance labels: source-grounded (read directly from a label or database), calculated (deterministically derived from source-grounded values), or model-inferred (estimated by computer vision, prediction, or a generative model). Model-inferred values are displayed with an uncertainty range and routed according to the risk tiers described in Section 9. Key terms are defined in Box 1.

Box 1 **Key computational terms used in this Perspective.**

Machine learning (ML): Methods that learn patterns from data to perform a task without explicitly programmed rules.

Prediction: An ML output estimating an unobserved or future quantity, such as risk of hyperkalemia, reported with its validation performance.

AI inference (model-inferred value): Any value or statement produced by a trained model rather than read from, or calculated deterministically from, a verified source.

Generative AI and large language models (LLMs): Models that generate text or other content; in DNCE they are used for capture, structuring, and explanation, not for computing nutrient values.

Retrieval-augmented generation (RAG): An LLM approach that retrieves passages from a defined, version-controlled document collection and grounds its response in them, with citations.

Deterministic calculation: A rule- or formula-based computation that returns the same output for the same verified input.

Source-grounded value: A value read directly from a nutrition label or food-composition database, with its source identified.

Table 2 summarizes the rationale for each component, with an illustrative example of how it may support patient safety, workflow efficiency, or professional oversight. DNCE is a conceptual framework; whether these components improve safety, efficiency, or outcomes in practice remains to be demonstrated empirically (Section 10).

## 6. Operationalizing DNCE: Dietitian Capacity in Support of Education and Care

One operational pathway is to map validated or emerging tools to functions rather than prescribe a single technology stack. Structured 24 h recall, AI-assisted food-image analysis, standardized nutrition documentation, speech-to-text (STT) or ambient scribing, and RAG provide complementary modules [15,21,22,23,24,25,26]. The Automated Multiple-Pass Method offers a validated structure for dietary recall but remains vulnerable to individual-level under-reporting [21]. Multimodal image analysis can accelerate food recognition and nutrient estimation but depends on representative data and reliable food-composition resources [22]. Recent work co-authored by Elío found that ChatGPT-5 image-based energy and macronutrient estimation improved when ingredient and preparation context was supplied and performed best against dietitian-annotated, curated reference data, reinforcing the need for context-rich inputs and expert confirmation [15]. Clinical speech recognition and ambient scribes can reduce documentation work, while still requiring review and correction [23,24]. RAG can retrieve curated evidence and integrate multiple data streams, but generated outputs remain provisional and require traceability and professional review [25,26].

Mapped to DNCE functions, AI-assisted food-image analysis supports data acquisition and intelligence and yields model-inferred values that require confirmation when they affect high-risk nutrients. Speech-to-text with LLM structuring supports documentation within nutrition diagnosis and clinical decision-making, producing a draft note that the dietitian edits and signs. Label-image extraction supports data acquisition, and its values become source-grounded once verified against the label. A rule-based nutrient engine supports data integration and MNT prescription with calculated values. RAG-generated education materials support delivery and engagement, with cited drafts reviewed before release.

The workflow logic is deliberately asymmetric: automation should be strongest where a task is repetitive, reversible, and easy to verify, while human involvement should increase as decisions become more consequential. Before a visit, the system may pre-populate diet histories, organize images, and generate preliminary nutrient summaries. During the encounter, ambient documentation may reduce clerical note-taking. After the visit, structured data and RAG may prepare a draft summary, surface missing information, retrieve relevant evidence, and assemble a first-pass care-plan scaffold. The dietitian can then concentrate professional attention on nutrition diagnosis, prioritization, goal negotiation, counseling, food implementation, and final approval. Figure 2 illustrates this division of labor and the workforce outcomes that should be tested.

Task allocation can follow explicit criteria. A task may be automated when it is deterministic or verifiable against a source, when errors are low in consequence and easily reversed, and when the required data are complete. Professional involvement is essential when a task requires clinical judgment; when errors could cause serious harm, as with electrolyte restrictions or insulin-related carbohydrate guidance; when data are missing or conflicting; when psychosocial or ethical issues arise, such as signs of disordered eating; or when the decision depends on the patient’s values and preferences.

The central practical claim is therefore not that AI makes nutrition care faster by itself, but that carefully orchestrated automation can reallocate professional time. Relevant outcomes include pre-visit review time, nutrient coding and calculation time, documentation time, correction and rework time, diagnosis-to-care-plan time, after-hours documentation, consultations completed per session, direct-care time, referral-to-consult waiting time, and cost per completed nutrition episode. Early indicators suitable for pilot studies include the proportion of AI drafts accepted without edits and the number and type of errors detected at professional review. A tool that produces a note in seconds but requires prolonged correction is not efficient; a modest tool that reliably saves several minutes at multiple workflow steps may create substantial capacity across a full clinic schedule.

The added value of generative AI should be demonstrated against simpler alternatives rather than assumed. For nutrient calculation from structured inputs, rule-based methods are preferable because they are transparent, reproducible, and inexpensive. Generative AI may add value mainly by converting unstructured inputs, such as speech, free text, and photographs of labels or meals, into structured data, and by producing individualized explanations. Evaluations should therefore include rule-based calculation and conventional structured extraction, such as optical character recognition with templates, as comparators, and should attribute benefit to generative AI only where it exceeds them on accuracy, time, or user outcomes.

## 7. An Empirical Starting Point: Why Nutrition-Label Verification Matters

A Taiwan-based study involving Lee’s Clinic provides a pragmatic reliability benchmark for DNCE task allocation [7]. Eight commercially available ready-to-eat meal boxes were independently estimated by four registered dietitians and repeatedly assessed from meal images by five conversational AI models, using product labels as the reference. Mean inter-dietitian coefficients of variation were 5.6% for energy, 14.3% for protein, 11.0% for carbohydrate, and 40.2% for sodium. Across AI models, intra-assay sodium variability ranged from 21.7% to 69.2%. Relative to product labels, dietitian estimates averaged 99.5% for energy and 91.8% for sodium, whereas no single AI model was consistently closest to the label across all nutrients [7].

The comparison used commercial product labels, not laboratory chemical analysis, as the reference; the findings therefore provide a reproducibility and label-concordance benchmark rather than proof of absolute nutrient validity. Declared label values are themselves permitted to deviate from actual content within regulatory tolerances, for example, under United States labeling rules [27]; accuracy studies should therefore use chemical analysis where feasible, or weighed food records combined with standardized recipes and food-composition databases for mixed dishes. Nor did the study test an educational intervention or the DNCE model. Its relevance to this Perspective is a clearly delineated educational problem: a plausible-looking AI estimate may differ from the printed label or change across repeated queries, while an unobserved ingredient or portion assumption may remain hidden. These observations motivate, but do not establish the efficacy of, a structured verification exercise. In clinical deployment, repeated-output testing, local food-composition grounding, model/version documentation, and distinct labels for “extracted from source” and “inferred by model” would serve both safety and education.

The comparison is also small and context-specific. With eight packaged ready-to-eat meal boxes from one market, it cannot be generalized to home-cooked or restaurant meals, street foods, beverages, or cuisines under-represented in model training data, nor to other languages, image conditions, or user groups. Future evaluations should use test sets stratified by food type, cuisine, language, image quality, and user group, including older adults and people with limited health literacy, and should report performance for each stratum, treating material differences between strata as potential bias to be addressed before deployment.

Nutrition-label verification is particularly useful for building critical AI nutrition literacy because the reference is visible to the learner. When an AI output can be checked against a label in hand, learners directly encounter errors that are otherwise hidden: invented values, confusion between per-serving and per-100 g figures, incorrect serving sizes, unit errors, and inappropriate rounding. Repeated practice can build a transferable habit of asking where a number came from and whether it can be verified, including in situations where no label exists. The exercise also teaches that references have limits, because declared values may differ from actual content. Learners thus move from accepting a single answer to judging the reliability of each source, which is the core of critical AI nutrition literacy.

## 8. Critical AI Nutrition Literacy: From Chatbot Answers to Verifiable Interpretation

The same reliability concerns that affect clinical workflow create a learning challenge. Chatbots may offer fluent explanations, formatted tables, and precise numbers even when serving size, ingredient identity, cooking method, or nutrient source has been inferred. Users therefore need to assess the provenance, assumptions, uncertainty, and consequences of an output—not merely recall basic nutritional facts. We propose treating verification as a learning objective in chatbot-supported education, in line with broader nutrition and food literacy principles [4,5,6]. Here, “misinformation” includes misleading or unsupported nutrition claims regardless of whether the error is intentional; the present article does not measure misinformation prevalence or demonstrate that the checklist reduces it.

We propose five questions (Table 3) for nutrition students, patients, consumers, and health professionals. First, is the value transcribed from a product label or authoritative database, or inferred by the model? Second, does the stated serving size match the edible quantity and number of servings? Third, which ingredients, preparation methods, and nutrient values were assumed, and what uncertainty remains? Fourth, is the output consistent across repeated attempts, and how does it compare with an authoritative source? Fifth, could acting on the answer affect health, indicating a need for dietitian or other qualified professional review? The checklist is proposed as a teaching and safety aid and has not yet undergone content validation, usability testing, or outcomes evaluation.

In brief, the checklist asks users to check five things before acting on an AI nutrition answer: the source of each value, the serving size, the assumptions made, the consistency of the output, and whether professional review is needed.

The educational goal shifts from “Can the chatbot answer the question?” to “Can the learner judge and appropriately act on the answer?” A proposed activity has learners compare a product label with one or more chatbot interpretations, mark which numbers were extracted versus inferred, explain any discrepancies, and state a verification or referral step. A brief teach-back can then ask patients to identify the serving size, point to the nutrient source, and describe when they would seek professional confirmation. Dietitians may integrate the checklist into educational encounters, intake workflows, or quality review. These are suggested teaching activities, not tested components of a validated curriculum.

A practical delivery pathway may involve a short educator demonstration, individual label-versus-chatbot comparison, peer discussion of discrepancies, and a teach-back or short-answer assessment. In a subsequent study, a standardized set of deliberately plausible but flawed AI outputs could be used to test whether learners identify errors without additional hints. Such materials would require prospective development, expert review, and safeguards for vulnerable patients; no such materials or outcomes are reported here.

Illustrative label-based teaching scenario (hypothetical, not study data). A learner uploads a photograph of a packaged ready-to-eat meal and asks a chatbot to estimate sodium and energy. The educator provides the original nutrition label and asks the learner to: (i) identify whether each chatbot value was read from the label or inferred from the photograph; (ii) check whether the chatbot assumed one serving or the whole package; (iii) list unobserved sauces or preparation assumptions; (iv) repeat the prompt and compare the response with the original label; and (v) explain whether a high-stakes dietary decision—for example, a clinically consequential sodium, allergen, or therapeutic dietary restriction—requires professional review. The expected educational endpoint is a justified decision about information reliability, not numerical agreement with the chatbot.

## 9. Risk-Tiered Human Oversight and Governance

Human-in-the-loop design should be operational rather than rhetorical. Low-risk functions—food logging, reminders, data extraction, trend visualization, transcription drafts, preliminary coding, routine calculations, or delivery of previously approved education—may be automated with quality controls. Intermediate-risk functions, such as identifying probable nutrition problems, suggesting meal substitutions, or drafting Problem–Etiology–Signs/Symptoms statements, should be reviewable, traceable, and easy to correct. High-risk nutrition decisions require qualified clinician confirmation, including major energy or protein prescriptions in malnutrition, refeeding-risk management, enteral or parenteral nutrition planning, clinically significant electrolyte restrictions, food-allergy advice, and recommendations with important medication–nutrient interactions.

Each tier also requires operational rules for uncertainty. First, each task should have a required-information schema; for example, advice on sodium or potassium restriction requires recent laboratory values, kidney function, and current medications, and a missing required field should prompt a query or abstention. Second, model-inferred values should be shown as ranges with their provenance label and a plain-language note, such as “estimated from photo; may differ substantially from the label”. Third, the system should abstain and refer to a dietitian or other qualified professional when required data are missing, the task falls in the high-risk tier, a model-inferred value lies near a clinical threshold, sources disagree, or the case is outside scope, such as signs of disordered eating, pregnancy, children, or dialysis. Finally, test sets should include cases in which the correct response is to abstain, and performance should be reported as appropriate- and unnecessary-abstention rates, risk–coverage curves, and the rate of potentially harmful recommendations.

This task-allocation model has a direct workforce rationale. In a systematic review of ambient AI-scribe implementation, nine of ten studies reporting efficiency outcomes showed improvement in at least one efficiency metric, and seven of ten reported positive effects on clinician wellness or burnout, although accuracy varied and manual editing remained common [24]. These findings come from broader clinical documentation workflows rather than dietetics alone, but they illustrate a central DNCE principle: automation creates useful capacity only when correction and rework are controlled.

Responsibility must remain explicit. Clinical responsibility should remain explicitly assigned to qualified professionals in accordance with local regulation and institutional governance. WHO guidance on AI for health and large multimodal models emphasizes ethics, accountability, transparency, safety, stakeholder involvement, and post-deployment oversight [28,29]. DNCE therefore requires audit trails, uncertainty signaling, escalation rules, the ability to contest or override algorithmic output, and educational interfaces that distinguish source-grounded facts from model inference.

Governance frameworks translate these principles into enforceable obligations. Beyond WHO guidance, the European Union Artificial Intelligence Act imposes risk management, data governance, human oversight, and post-market monitoring obligations on high-risk systems, including AI that is part of a medical device [30]. United States guidance on clinical decision support software treats transparency as a key criterion: health professionals should be able to independently review the basis of a recommendation [31], a requirement that DNCE provenance labels are designed to meet. In DNCE, these principles become five mechanisms: clear allocation of responsibility among the developer, the deploying institution, and the reviewing professional; registration and change control for every model and prompt version; an audit trail linking each recommendation to its sources and reviewer; incident reporting for AI-related errors and near misses; and post-deployment monitoring of performance across patient groups.

To support these mechanisms, each value should carry a minimum provenance record: its provenance label; source identification, such as the food-composition database name, version, and food code, or the stored label image; timestamps for capture and computation; for model-inferred values, the model name and version, prompt-template version, and retrieval-corpus version; and the reviewing professional and the action taken (accepted, edited, or rejected). These elements map onto the HL7 FHIR Provenance resource, allowing them to travel with the data into electronic health records [32].

## 10. Evaluation, Education Outcomes, and Research Agenda

DNCE should be evaluated as an educational, clinical, and workforce intervention rather than solely as a model-accuracy benchmark. Future work could begin with expert content review and usability testing of the five-question checklist, followed by feasibility studies and controlled comparisons of checklist-supported instruction versus usual nutrition-label education. Early-stage clinical AI evaluation may draw on DECIDE-AI [33]; prediction-model reporting on TRIPOD+AI [34]; randomized AI interventions on CONSORT-AI [35]; and large language model studies on TRIPOD-LLM [36] where applicable. Implementation outcomes may be organized using RE-AIM [37] and the updated Consolidated Framework for Implementation Research [38]. These frameworks should be selected according to the actual study design, not assumed to apply to every component.

Table 4 outlines a phased research plan with separate validation pathways for the checklist, as an educational instrument, and for DNCE, as a care model. Each phase has a prespecified criterion for progressing to the next phase.

Evaluation should explicitly distinguish learning, behavior, clinical safety, and workforce effects. Four domains should be kept separate: technical model performance (are outputs accurate and reproducible?), educational effectiveness (can learners appraise AI outputs?), clinical safety (does use avoid harm?), and workflow efficiency (does it free professional time for judgment-intensive care?). Proposed primary educational outcomes include correct identification of label-extracted versus AI-inferred information, recognition of serving-size mismatches and unsupported assumptions, appropriate verification against source labels, and identification of situations requiring professional review. Possible secondary outcomes include learner confidence calibrated against correctness, retention of verification behavior, response to deliberately inconsistent AI outputs, and teach-back performance. Predefined scoring rubrics, blinded assessment when feasible, and testing across age, language, and health-literacy groups would be useful. Data-acquisition and documentation outcomes should include missingness, nutrient estimation error (reported as mean absolute error, root mean squared error, mean absolute percentage error, Bland–Altman limits of agreement [40], Lin’s concordance correlation coefficient [41], and the proportion of estimates within ±10% and ±20% of the reference), repeated-input variability (intraclass correlation coefficient and coefficient of variation across repeated runs), omissions or fabrications, correction time, and version drift. Care and implementation outcomes should include prescription-to-consumption fidelity, comprehension, engagement, direct-care time, net workload after corrections, access, costs, equity, and sustainability. Model performance alone should not be presented as evidence of educational benefit.

Robustness should be tested along five axes: repeated identical prompts, with several runs per input; model versions, with benchmark re-runs as regression tests whenever a model is updated; language, comparing equivalent inputs in Traditional Chinese and English and including local dish names; image quality, using standardized variations in lighting, angle, blur, occlusion, and resolution; and missing information, by systematically withholding portion size, cooking method, or clinical parameters. Consistent with TRIPOD-LLM [36], studies should report the model name, version or snapshot identifier, access date, and interface; inference parameters such as temperature; full system prompts and prompt templates as supplementary material; and the version of any retrieval corpus.

The framework has important boundaries. DNCE is conceptual and has not yet demonstrated incremental effectiveness over well-implemented NCP or other integrated-care models. Infrastructure, interoperability engineering, cybersecurity, data governance, and workforce training can create substantial costs. Automation can also generate new work through correction, alert fatigue, duplicate documentation, or version instability. Digital exclusion related to age, language, disability, connectivity, and digital literacy may increase burden if equity is not designed into the workflow.

To isolate incremental value, future studies should compare the proposed education checklist with conventional label-reading instruction and, separately, distinguish any additional benefit from embedding it within the broader DNCE workflow. Pre–post gains alone would not establish causation; randomized or controlled designs, where feasible, could test transfer to unfamiliar food labels and AI responses, durability of learning, and potential unintended effects.

Food implementation is a further boundary. More rapidly generated recommendations do not ensure that prescribed food can be accessed, afforded, prepared, accepted, or consumed. Controlled Food Is Medicine research cautions against equating access or participation with clinical benefit [42], and implementation scholarship highlights financing and integration with real-world services [13]. DNCE’s proposed educational element also requires careful testing: learners might become more confident without becoming more accurate; repeated chatbot interrogation could introduce additional misinformation; and verification burdens may fall unevenly on people with limited literacy, disability, language access, or connectivity. Prospective evaluations should test whether the framework improves verified understanding and appropriate escalation without increasing inequity or total workload.

## 11. Conclusions

Digital innovation in nutrition education should be judged not by the fluency or number of automated outputs, but by whether people can interpret nutrition information accurately and act on it safely. DNCE prioritizes human oversight, source verification, and practical educational tools to counter unsupported AI nutrition claims. DNCE proposes a human-centered link between Nutrition Care Process-aligned services and critical AI nutrition literacy: professionals oversee consequential decisions; verified source data are distinguished from model inference; food recommendations are connected to feasible implementation; and learner and care outcomes feed back into the system. Its five-question checklist offers a promising but unvalidated starting point for teaching source checking, serving-size verification, assumption recognition, consistency testing, and appropriate escalation. The central research proposition is that a dietitian-led, label-centered educational approach embedded in a coordinated digital workflow may improve resistance to unsupported AI nutrition claims while preserving safety, equity, and professional capacity. This claim remains to be tested: empirical studies, progressing from usability testing to controlled trials, are needed to confirm the checklist’s effectiveness and the framework’s impact on safety, equity, and professional capacity.

## Figures and Tables

**Figure 1:**
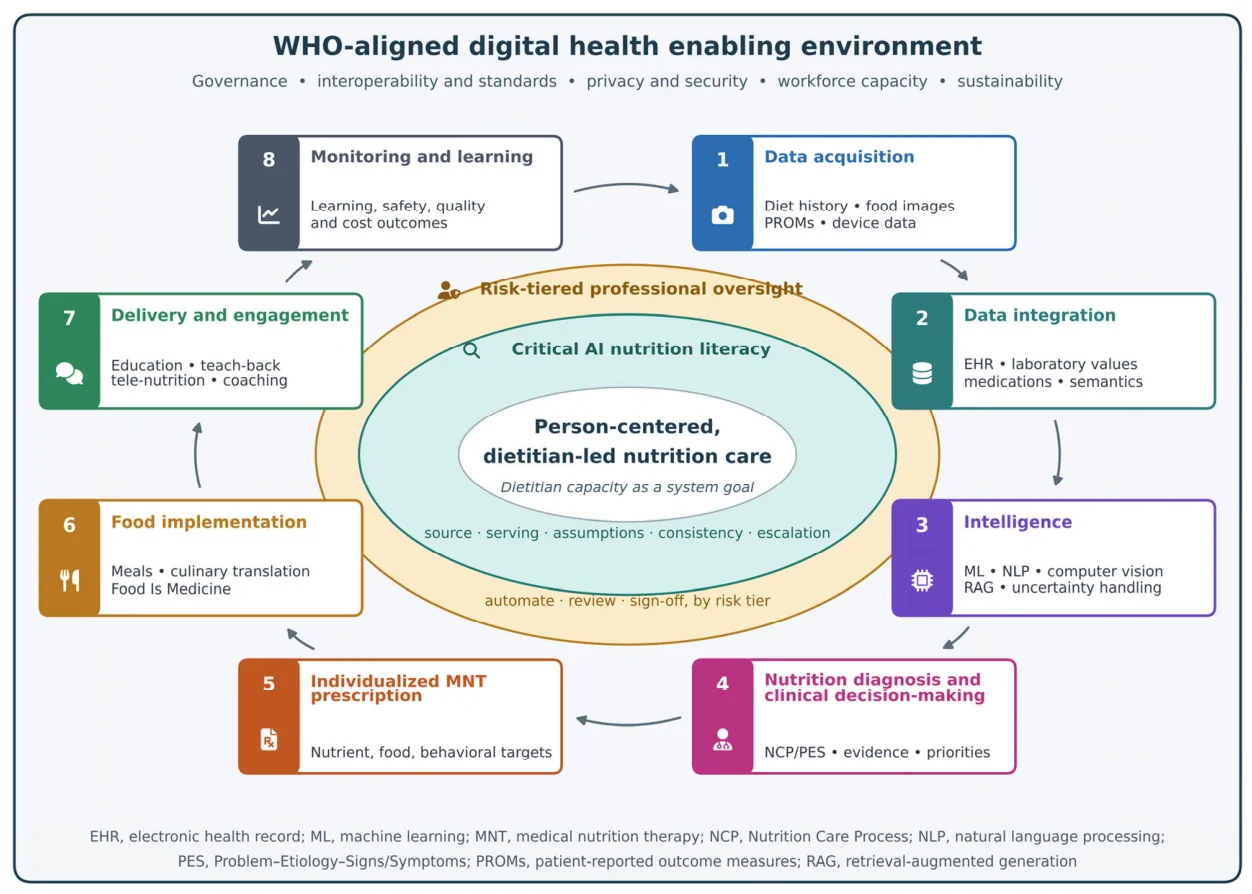
Digital Nutrition Care Ecosystem closed-loop framework showing eight nutrition-care components, critical AI nutrition literacy, dietitian-led oversight, and a WHO-aligned enabling environment. Colors distinguish the eight components; the two inner bands show critical AI nutrition literacy and risk-tiered professional oversight, which apply to every component. Arrows indicate the direction of the closed care loop, from data acquisition (1) through monitoring and learning (8) and back to data acquisition; icons are illustrative symbols for each component and band. The diagram depicts the underlying care infrastructure and dietitian capacity as a system-level goal; this Perspective extends its interpretation by specifying critical AI nutrition literacy as a cross-cutting educational requirement at the points where inferred data are translated into patient-facing information, verified, and revisited through monitoring. The figure is a conceptual model, not evidence of intervention effectiveness. Abbreviations: AI, artificial intelligence; EHR, electronic health record; ML, machine learning; MNT, medical nutrition therapy; NCP, Nutrition Care Process; NLP, natural language processing; PES, Problem–Etiology–Signs/Symptoms; PROMs, patient-reported outcome measures; RAG, retrieval-augmented generation; WHO, World Health Organization.

**Figure 2:**
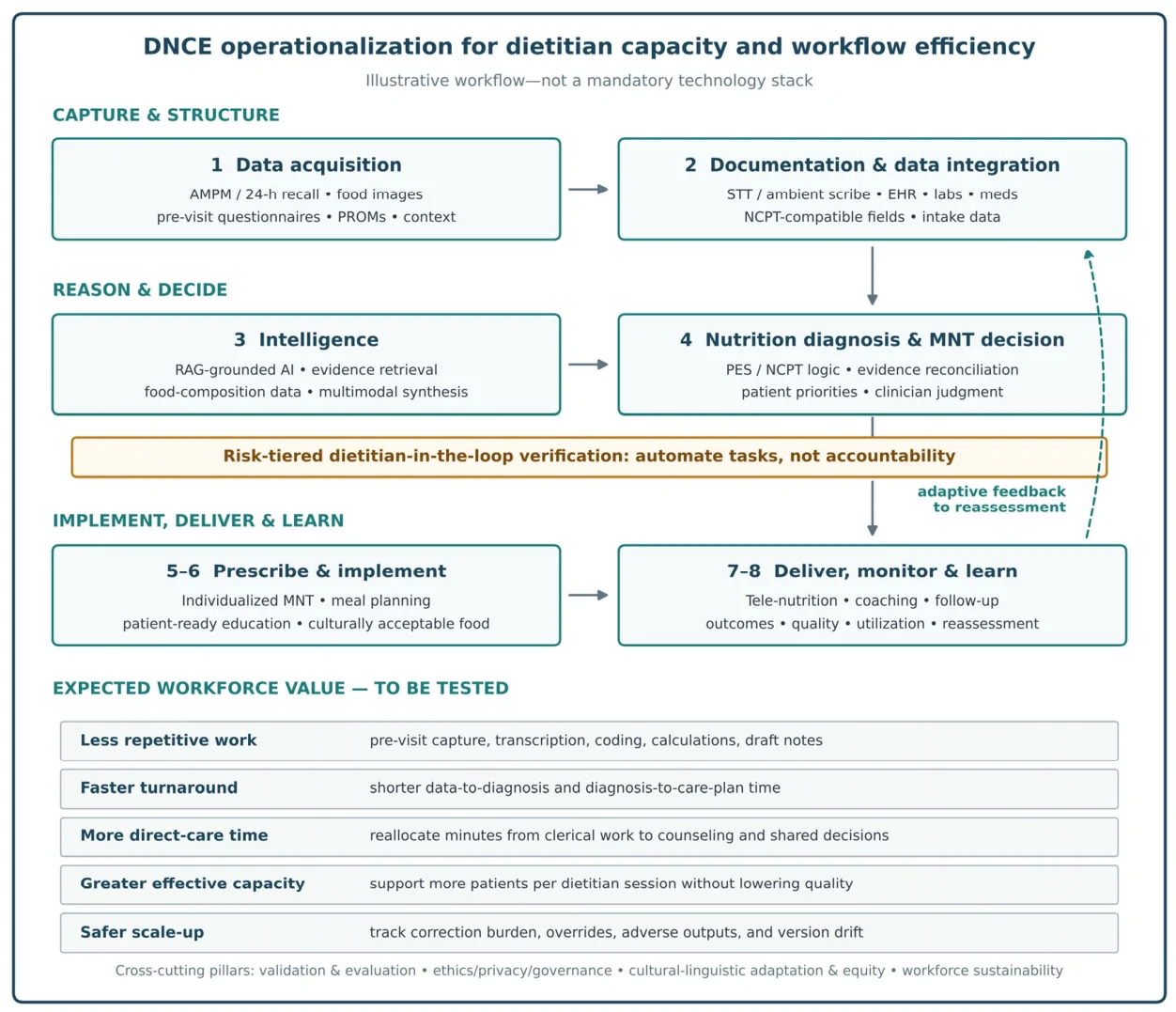
Illustrative operationalization of DNCE for clinical workflow and dietitian capacity. The figure distinguishes digital support for intake, transcription, evidence retrieval, and drafting from clinician-controlled diagnosis, MNT decisions, and final approval. In the proposed education application, time recovered from repetitive tasks could be redirected toward guided verification of AI nutrition claims, teach-back, and shared decisions; this reassignment and its outcomes require prospective testing. The modules illustrate one possible pathway, not a required technology configuration. Solid arrows indicate the sequence of workflow steps; the dashed arrow indicates adaptive feedback from delivery and monitoring to reassessment. Abbreviations: AI, artificial intelligence; AMPM, Automated Multiple-Pass Method; DNCE, Digital Nutrition Care Ecosystem; EHR, electronic health record; MNT, medical nutrition therapy; NCPT, Nutrition Care Process Terminology; PES, Problem–Etiology–Signs/Symptoms; PROMs, patient-reported outcome measures; RAG, retrieval-augmented generation; STT, speech-to-text.

**Table 1:** Comparison of existing categories of AI-supported nutrition tools with the proposed DNCE.

Approach	Source of Nutrient Values	Provenance Shown to User	Uncertainty and Abstention	Professional Oversight	Link to Food Implementation and Outcomes
Rule-based nutrient calculators and dietary assessment software	Verified food-composition databases	Database named; item-level source rarely shown	Point estimates; no abstention	Depends on user	Absent
Image-based food recognition apps	Computer-vision estimates of food identity and portion	Rarely shown	Confidence seldom shown	Usually absent	Absent
General-purpose chatbots	Model-generated; sources often unstated	Rarely shown	Inconsistent; seldom abstain	Absent	Absent
RAG-grounded assistants	Retrieved documents plus generated text	Citations to retrieved documents	Limited	Variable	Rare
EHR-integrated clinical decision support	Structured EHR data and rules	Within EHR audit trail	Alert-based	Clinician-facing	Rarely reaches food level
DNCE (proposed)	Deterministic calculation from verified sources; AI for capture, extraction, and explanation	Each value labeled source-grounded, calculated, or model-inferred	Missing-information checks, abstention, and escalation	Risk-tiered dietitian review	Closed loop to food implementation and outcome monitoring

Note: Categories are illustrative; individual products may differ. Abbreviations: AI, artificial intelligence; DNCE, Digital Nutrition Care Ecosystem; EHR, electronic health record; RAG, retrieval-augmented generation.

**Table 2:** Rationale and illustrative examples for the eight DNCE components.

Component	Rationale	Illustrative Example	Main Contribution
1. Data acquisition	Capture real-world data with minimal burden	Meal photographs and home glucose records collected before the visit	Workflow efficiency
2. Data integration	Create one longitudinal, interoperable record	Intake displayed alongside laboratory values and medications	Patient safety
3. Intelligence	Use AI within bounded, labeled roles	Portion estimates labeled model-inferred; draft summaries for review	Efficiency; transparency
4. Nutrition diagnosis and clinical decision-making	Keep professional reasoning central	Dietitian confirms the PES statement and sets nutrient targets	Professional oversight
5. Individualized MNT prescription	Tailor targets to the person	Sodium and protein targets adjusted for kidney function and preferences	Patient safety
6. Food implementation	Translate the prescription to the plate	Teaching-kitchen session on lower-sodium choices among ready-to-eat meals	Adherence
7. Delivery and engagement	Sustain participation without adding burden	Teach-back using the five-question checklist	Engagement; AI literacy
8. Monitoring and learning	Feed outcomes back into care and education	Review of intake, laboratory trends, and correction logs to refine rules	Safety; learning

Note: Examples are illustrative and have not been evaluated. Abbreviations: AI, artificial intelligence; MNT, medical nutrition therapy; PES, Problem–Etiology–Signs/Symptoms.

**Table 3:** Five-question critical appraisal checklist for chatbot interpretations of nutrition information and nutrition labels.

Critical Appraisal Question	Why It Matters	User/Learner Action	Dietitian/System Safeguard
1. Source	Is the nutrient value or interpretation directly extracted from a product label or authoritative database, or inferred by the chatbot?	Ask the system to identify the source and compare it with the original label/database.	Preserve provenance; label-extracted versus inferred fields; link to authoritative sources when available.
2. Serving size/portion	A correct nutrient value can still be misleading if the portion assumption is wrong.	Confirm serving size, edible portion, number of servings, and whether the image represents the whole meal.	Require explicit portion assumptions and make serving-size edits easy.
3. Assumptions and uncertainty	Hidden oils, sauces, mixed dishes, preparation methods, and missing ingredients can materially change estimates.	Ask what was assumed and which values are uncertain; avoid treating precise numbers as measured values.	Display uncertainty, missing information, and assumptions; trigger review when key inputs are absent.
4. Consistency and verification	Generative outputs may vary across repeated prompts, model versions, or languages.	Repeat the query when precision matters and cross-check clinically important values against a reliable source.	Track model/version; perform repeated-input testing; monitor correction burden and drift.
5. Escalation and action	The importance of an error depends on what will be done with the answer.	Seek professional review before using AI output for consequential diet prescriptions or therapeutic decisions.	Apply risk-tiered dietitian sign-off rules and maintain an audit trail for overrides and final release.

Note: The checklist is intended as an educational and quality-improvement aid, not as a substitute for professional clinical judgment. Abbreviations: AI, artificial intelligence.

**Table 4:** Phased research plan with separate validation pathways for the checklist and the DNCE framework.

Phase	Checklist Pathway (Educational Instrument)	DNCE Pathway (Care Model)	Example Outcomes	Criterion to Progress
1. Content review and usability	Modified Delphi with dietitians, educators, and physicians; cognitive interviews with learners and patients	Think-aloud usability testing with dietitians and patients	Content validity index [39]; System Usability Scale	Content validity and usability above prespecified thresholds
2. Reliability and technical benchmarking	Inter-rater agreement when applying the checklist to standardized AI outputs	Stratified test sets with reference standards; robustness testing	Kappa; MAE, RMSE, Bland–Altman limits, ICC; abstention performance	Error within prespecified tolerance across strata
3. Feasibility	Pilot teaching sessions	Retrospective case simulation and single-site prospective pilot with safety monitoring, reported per DECIDE-AI [33]	Time–motion data; errors detected at review; safety events	No unresolved safety signals; feasibility targets met
4. Controlled comparison	Randomized comparison with conventional label-reading instruction	Pragmatic cluster-randomized or stepped-wedge comparison with usual dietetic workflow	Error-detection accuracy; direct-care time; clinical outcomes	Effect on prespecified primary outcome
5. Implementation	Multi-site use in curricula	Multi-site implementation	RE-AIM dimensions [37]; costs; equity across subgroups	—

Abbreviations: AI, artificial intelligence; DECIDE-AI, Developmental and Exploratory Clinical Investigations of Decision support systems driven by Artificial Intelligence; DNCE, Digital Nutrition Care Ecosystem; ICC, intraclass correlation coefficient; MAE, mean absolute error; RE-AIM, Reach, Effectiveness, Adoption, Implementation, Maintenance; RMSE, root mean squared error.

## Data Availability

No new data were created or analyzed in this study. Data sharing is not applicable to this article.
